# 3-D Evaluation of temporary skeletal anchorage sites in the maxilla

**DOI:** 10.4317/jced.57574

**Published:** 2021-11-01

**Authors:** Humberto Zago, Ricardo-de Lima Navarro, Vinicius Laranjeira, Thais-Maria-Freire Fernandes, Ana-Cláudia-de Castro-Ferreira Conti, Paula-Vanessa-Pedron Oltramari

**Affiliations:** 1 DDS, MSc. Former Graduate Student, Department of Orthodontics, UNOPAR - University of North Paraná, Brazil; 2 DDS, MSc, PhD. Private practice, Londrina, Paraná, Brazil; 3 DDS, MSc, PhD. Private practice, Joinville, Santa Catarina, Brazil; 4 DDS, MSc, PhD. Assistant Professor, Department of Orthodontics, UNOPAR - University of North Paraná, Brazil

## Abstract

**Background:**

The selection of temporary anchorage device (TAD) site can be a challenging task since one should not only consider the 2-D distances between roots, but also the entire 3-D space. Thus, the aim of this study was to evaluate the posterior maxillary region areas available for the insertion of temporary anchorage devices in reconstructed images from cone beam computed tomography (CBCT).

**Material and Methods:**

Sample consisted of 72 patients with indication for orthodontic treatment, grouped into three distinct age groups: 11 to 14 years (age group 1), 15 to 19 years (age group 2) and aged 20 years or older (age group 3), which were further subdivided as to the type of malocclusion (Angle Class I, II and III). Orthopantomographic reconstructions and cross sections were obtained with the Dolphin Imaging software. The distance between the roots of maxillary teeth (canines, premolars and first molars) was determined at 5 mm of the cementoenamel junction, as well as the depth of bone availability at different insertion angles (90°, 75°, 60°, 45°). The influence of different angulations, age, and malocclusion on bone availability was evaluated by ANOVA, followed by the Bonferroni post-test. For the evaluation of the interaction of these factors, 2-way ANOVA was used.

**Results:**

Bone availability was found to be poor between roots in the molar region. There was a reduction in bone availability with increasing age. With regard to angulations, greater bone availability was found in depth for 45° angulation in the canine and first premolar regions and for angulation of 75° or 90° in the molar region. However, there was no difference between bone availability in the region of the second premolars.

**Conclusions:**

According to applied methodology it can be concluded that the region between canines and premolars accepts better vertical angular variations for TADs insertion.

** Key words:**Cone-Beam Computed Tomography, Orthodontic Anchorage Procedures.

## Introduction

Anchoring as resistance to undesired displacement of one or more dental elements has always been a challenge for Orthodontics. The use of conventional anchorage with extraoral appliances often becomes impracticable due to oral conditions, aesthetic expectations, and especially patient compliance (1,2) and factors that can minimize these effects have been studied (2-6). The determination of a device that ensures tooth movement, with minimal side effects, is fundamental in the evolution of skeletal anchorage mechanics (4,7).

The use of devices as temporary anchorage devices (TADs) is increasingly frequent, as it facilitates orthodontic treatment and increases clinical possibilities (2,8-10). Its main advantages are related to the reduced size of the devices, which increases the areas available for installation, low cost, do not depend on the patient’s compliance and are easy to install and remove (1,2,5,8,9,11-13).

The success rate of devices ranges from about 60 to 100% (2,11,14,15). The considerable percentage of failure has been the reason for several studies that seek to determine risk factors for the success of devices. As results, several factors are often pointed out as potentially capable of influencing the stability of devices. Among these factors are insertion site (2,16), insertion angle (16,17), length and diameter(18,19); and the possibility of injuring adjacent structures (8,11,12,20). In addition, some recent studies have considered bone availability as a relevant risk factor for the failure of this anchoring system (1,10,18,21-23). However, contradictory results regarding the degree of influence of these various factors on the success rate of the devices are constantly observed in the literature, due to the heterogeneity of the samples and a large number of variables studied.

Due to the development of Cone Beam Computed Tomography (CBCT) the evaluation of three-dimensional areas available for the placement of the devices has become possible in a more secure way (7,24-26). This evaluation can be performed by measuring cortical thickness, the distance between cortical (buccal and palatal), distance from cortical bone to root, interproximal space of teeth and space between the roots of the posterior teeth (7,25).

Despite the constant improvements in mini-implant technology (27) and the benefits brought by more accurate examinations, this technology is not yet accessible to many clinicians due to learning difficulties, lack of training (12,21,28) and especially to the high cost of 3-D examinations and specific software (29). In addition, most treatments that include the use of TADs are performed based on 2-D location examinations, which provide a limited anatomical map of the most propitious regions and their best insertion angles (30).

The selection of the location of the skeletal anchoring device can be a challenge since one should not only consider the 2-D distances between roots but also the entire 3-D space (5,11), biological spaces and the adjacent structures (roots, nerves, vessels or air spaces) (31). Considering the lack of 3-D studies on bone availability for the installation of TADs, the objective of this study is to analyze in the CBCT the areas available for the installation of devices, as well as the possibility of variations in the angle of TAD insertion.

## Material and Methods

Institutional Review Board of the University of North Parana (UNOPAR), Londrina, Brazil approved this study.

Seventy-two CBCT images of patients without previous orthodontic treatment were analyzed. The tomographic images were generated using i-Cat (Imaging Sciences, Kavo, Protocol: 22x16 cm fov, 40 sec, 0.4 voxel, 120 KVP, and 36 mA), with 0.4 mm slices. The generated images were exported to the Dolphin software in DICOM format. The individuals were grouped according to age range (11 to 14 years, 15 to 19 years and over 20 years), which were further subdivided as to the type of malocclusion (Angle Class I, II and III) and sex distribution. Inclusion criteria were: the presence of all permanent teeth or their germs in the irruption phase (except third molars), the absence of previous orthodontic treatment, the absence of periapical pathologies and orthodontic treatment need.

**Figure 1:**
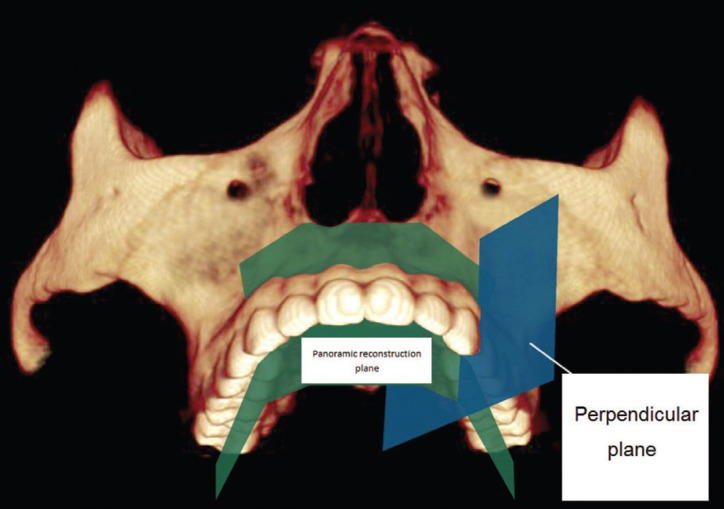
Tomographic image scheme simulating the panoramic reconstruction plane (green) and the plane perpendicular to the alveolar ridge (blue).

**Figure 2:**
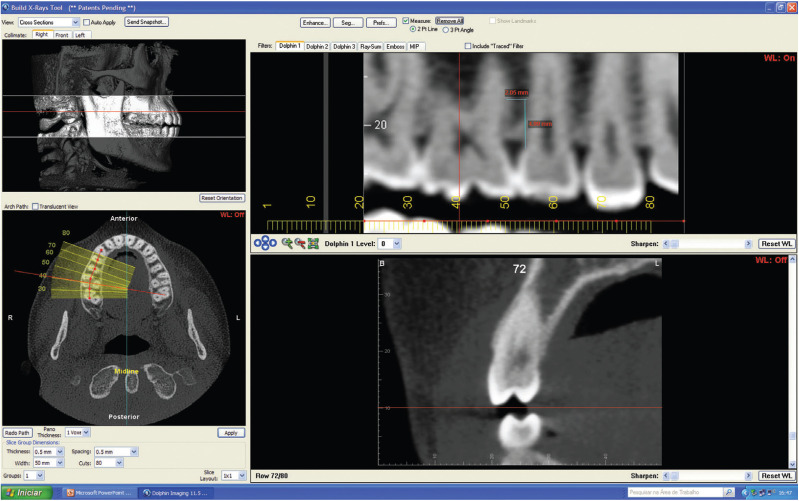
Image obtained from the Dolphin imaging software 11.5® with the sectional reconstruction tool of the alveolar ridge in 4 views (pictures) available in this feature: 1st left upper frame - lateral skull for selection of limits to be reconstructed (white lines) and delimitation of the position of the axial plane (red line); 2nd lower left frame - axial view for delimitation of the panoramic reconstruction reference line and below it the parameters for definition of reconstructed images (pan thickness, reconstruction thickness, reconstruction spacing, reconstruction width and reconstructed images); 3rd right upper frame - panoramic image reconstructed with markings (in blue) of the measurements height of 5mm of the cementoenamel junction and space between the roots at this height; 4th right lower frame - reconstruction image perpendicular to the alveolar ridge 0.5 mm thick.

A panoramic (orthopantomographic) reconstruction of areas between roots of posterior maxillary teeth was obtained through a 3-D visualization and the analysis was performed by means of using Dolphin Imaging 11.5 software (Patherson, Chatsworth, Calif.) (Fig. 1). These areas were evidenced on both sides, determined at a distance of 5 mm from the cementoenamel junction (2,17,32), where measurements were performed between the roots (Fig. 2).

**Figure 3:**
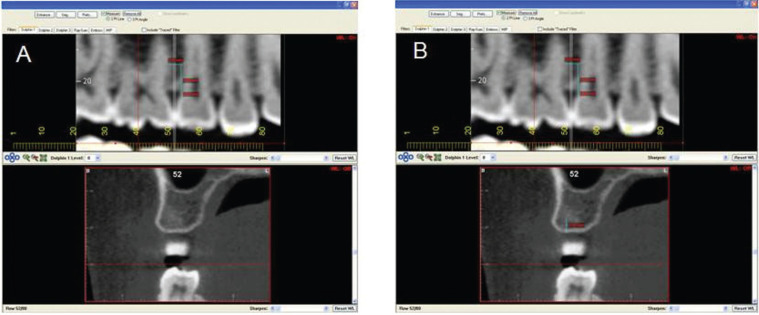
Transfer of the cementoenamel junction from reconstructed panoramic images to the sectional perpendicular to the alveolar ridge: A) measurement of the cementoenamel junction to the alveolar crest in the panoramic image; B) Transfer of the distance measured in the panoramic image to the sectional image perpendicular to the alveolar ridge (5 mm subtracted from the distance between the cementoenamel junction and the alveolar crest).

In addition to the distances between roots, the available depths were quantified. For this, sectional reconstruction images of 0.5 mm perpendicular to the alveolar ridge were obtained at the midpoint between the roots. In order to obtain the insertion point in the sectional image, it was necessary to transfer the location of the cementoenamel junction, observed only in the panoramic view (Fig. 3).

**Figure 4:**
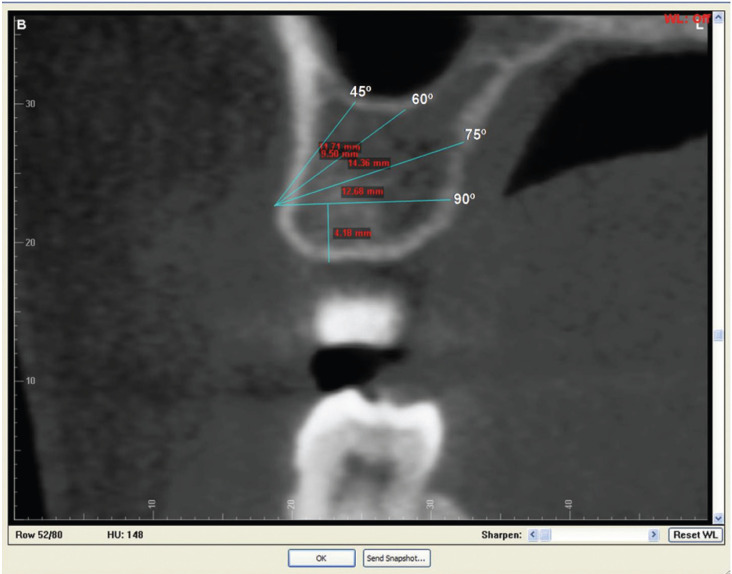
Simulation of the different insertion angles (90º, 75º, 60º, 45º) in the transferred position of 5mm from the cementoenamel junction.

After the location of the insertion point in the sectional image, different insertion angles (90°, 75°, 60°, 45°) and depth availability of the device was analyzed (Fig. 4).

-Statistical analyses

Only one previously calibrated investigator performed all measurements to avoid the interexaminer error. To evaluate the intraexaminer error, measurements of 20 randomly selected patients were repeated with a mean interval of 30 days to evaluate the systematic (paired t-test) and random error (Dahlberg’s formula).

After the Shapiro-Wilk test, mean and standard deviation parameters were used. One-way ANOVA followed by Bonferroni post-test evaluated the influence of different angulations, age range and type of malocclusion on bone availability. In order to evaluate the possible effect of the interaction of these factors on bone availability, two-way ANOVA analysis was performed. Sex and malocclusion distribution was assessed by the Chi-squared test.

Statistical analysis was performed using GraphPad Prism 5.0, BioEstat 5.0 and G Power 3.0 programs. A 95% confidence interval and a significance level of 5% were adopted for all tests applied.

## Results

Regarding the systematic error, a difference of less than 5% between the two measurements was observed, except for the canine region. However, no random error was observed.

Similar sex distribution was observed in the different age groups (Table 1).

**Table 1:**
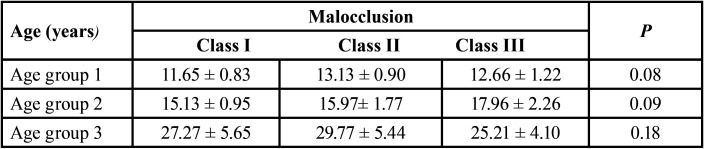
Distribution of the calculated age in relation to the type of malocclusion in the different age groups of the study.

**Table 2:**
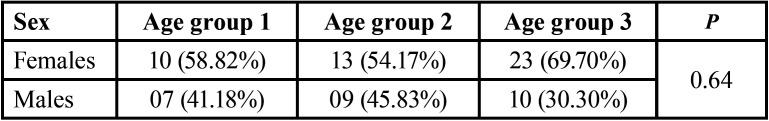
Sex Distribution (absolute and relative frequency) in the different age groups of the study (Chi-square test).

Bone availability between roots in the canine region (mean: 2.58 ± 0.74) was similar to that observed in the region of first premolars (Mean: 2.51 ± 0.70) and second premolars (Mean: 2.47 ± 0.93). However, lower bone availability was observed in the first molar region (Mean: 1.76 ± 0.72), according to the analysis of variance (repeated measures ANOVA, *p*<0.0001, the power of the test: 0.95, Fig. 1).

The type of malocclusion and age did not influence bone availability, except for the canine region ( Table 2, Table 3).

**Table 3:**
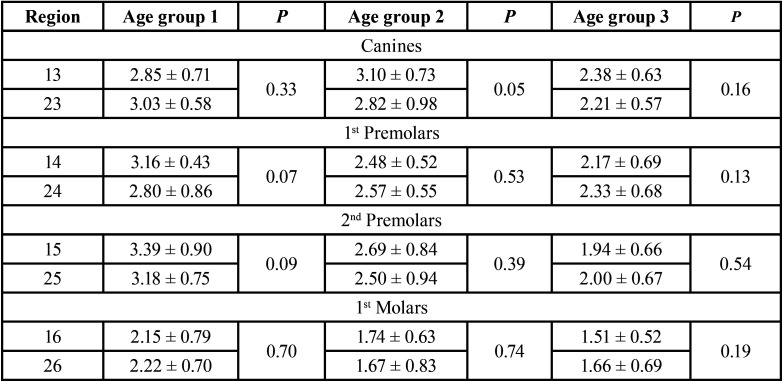
Evaluation of bone availability in mm (mean ± SD) in different regions of the maxilla in the different age groups comparing the right and left side.

Angulation affects bone depth availability independently in all age groups (*p*=0.0001), except for the region of second premolars in the age group 1. In addition, age also interferes with bone availability independent of angulation (*p*=0.0001). On the other hand, it was observed that the interaction between age and angulation affects bone availability in depth in the region of first premolars (*p*=0.02), according to the two-criteria analysis of variance ( Table 4- Table 7).

**Table 4:**
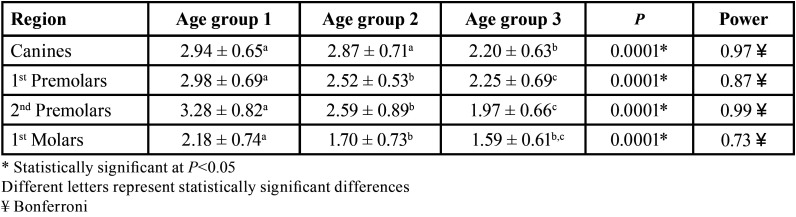
Evaluation of bone availability in mm (mean ± SD), in different regions of the maxilla with different age groups.

**Table 5:**
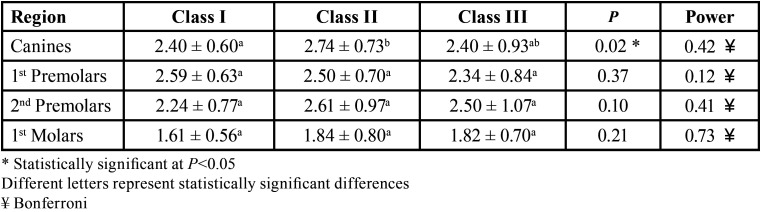
Evaluation of bone availability in mm (mean ± SD), in different regions of the maxilla with different age groups.

**Table 6:**
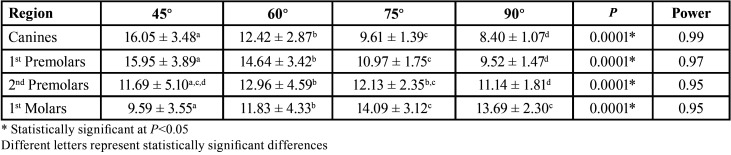
Evaluation of bone availability in depth in mm (mean ± SD) and in different regions and with different angulations.

**Table 7:**
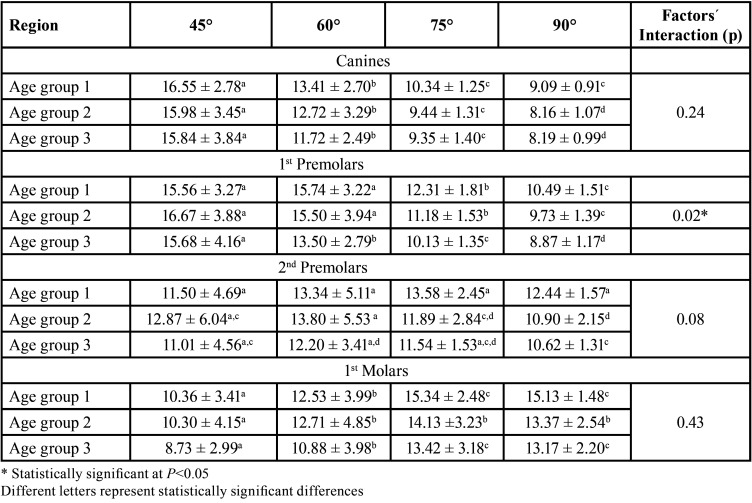
Evaluation of bone availability in depth in mm (mean ± SD) and in different regions and with different angulations in the different age groups.

## Discussion

The devices promote anchorage stable enough to perform the orthodontic movements, as long as they remain stable, without causing insults to the adjacent structures (8,11,12,33). However, the loss of the mini-implant during orthodontic treatment, before the end of its function, has been a significant event and it damages orthodontic mechanics. This data suggests the need to evaluate several variables during the technical planning for its installation (5,11,20,25), since few studies have evaluated the availability of bone three-dimensional and with significant samples (5,16,31,34). In addition, overall success rates for devices range from 60% to 100%, with an average success rate of 80% and have the advantage of simple surgical application (8,11). However, these authors pointed out that increasing diameter and length increases success rates, but unfortunately also increases the risk of root damage during insertion of TADs, thus corroborating the premise of the need to perform good planning of insertion sites of these devices (8,11,18,35).

Successful use of TADs requires good surgical planning, considering anatomical repairs in 3-D form, especially dental roots and intercortical distances (23). In this way, the CBCT has been an important tool for studies and diagnosis of bone availability for the installation of TADs (16,18,23,29). However, the high cost and lack of access to CBCTs restrict their application, causing recent publications to have reduced samples (17,36,37).

Lemieux (18) investigated a better pattern for placement and also factors that could influence the stability of TADs, such as depth, insertion site, and bone density. Thus, it indicates that for greater success in the installation and therefore better anchorage, the combination of these factors is needed. He concluded that a bolt with the greatest possible depth without damaging the structures would be the best choice. However, a case-by-case study and CBCT-assisted planning are fundamental to the success of the treatment, and studies that use CBCT to identify the protocol of the best areas would be necessary, justifying the accomplishment of this study.

The sample of this study consisted of 72 CBCT from patients with orthodontic treatment need, who were divided by different age groups: 11 to 14 years, 15 to 19 years and over 20 years, and subdivided in relation to malocclusion. The division of the groups was carried out in order to systematically evaluate the influence of the increase of age on the bone availability of the evaluated areas since it has already been reported that there is a bone decrease with advancing age (38). In addition, the sample presented homogeneity in its distribution, with respect to gender and malocclusion. This care was taken with the composition of the sample, but there are already works demonstrating that the gender does not is associated with decreased stability or proportion of success of devices (16,36).

The posterior region of the dental arches is the region commonly chosen for the installation of the devices used as a direct anchorage feature in the anterior retraction. The posterior region of the maxilla is considered adequate for the insertion of these devices (1,16,22,36,39).

The Dolphin imaging 11.5 software (Patherson, Chatsworth, Calif) was used to identify areas between roots of posterior teeth in panoramic (orthopantomographic) reconstructions. On both sides, these areas were evidenced, 5 mm from the cementoenamel junction (1,2,16,17,32,35). The cementoenamel junction was used as a reference point for the identification of the region to be measured, unlike other studies that used the alveolar ridge as a reference (5,12). This reference was used, since it is easily identifiable clinically, it does not have interferences of periodontal problems as it happens with the alveolar crest, and can be visualized in a simpler way in the image similar to panoramic and in the orthogonal cuts generated from the CBCT. The distance of 5 mm chosen is in accordance with previous studies because it is a favorable area to the installation of TADs due to proximity to the mucogingival line (2,5,35,40).

There was no statistically significant difference in bone availability between the roots when comparing the right and left sides of the same patient in the regions studied, corroborating Kim *et al*. (35). In this way, the data were grouped in relation to the region of the dental groups to facilitate the analysis of the data.

The bone availability between roots in the canine region was similar to that observed in the region of first and second premolars as previously found (36). However, in this study, significantly lower bone availability was observed in the first molar region (Fig. 1). It is suggested that this lower bone availability between first and second molars is related to the vertical height chosen to simulate the insertion of the devices. Other authors (16,31) also reported a trend of decrease in mesiodistal distance between the roots at approximately 6 mm of the cementoenamel junction, with values similar to those found in this study.

A new cut (or section) of tomographic reconstructions (23) was necessary for the evaluation of bone availability in depth. Care was taken at the time of transfer of these images so as not to lose the reference of the same evaluated sites when measuring the distances between roots. This new evaluation becomes relevant because the literature presents variations in the angle of insertion of the TADs (2,5,35,40). The variation in the angle of insertion to a more apical region can be indicated to not reach roots with greater proximity and at the same time to maintain its insertion in keratinized mucosa, more appropriate for the success of the screws (2,5,35,40). In addition, some authors report that the variation in insertion angle may also increase contact with bone tissue, increasing the resistance of the devices and this increased bone availability allows the use of devices with longer lengths (12,16,17,36,41). However, in spite of the greater anchorage with long devices, they are more susceptible to breakage during removal (18) and present a greater risk of damage to adjacent structures due to the longer length (8,11,18,35).

In this study, greater availability was observed in the canine and first premolar regions, at 45° angulation and a decreasing tendency of bone availability with increased angulation. In the second premolar region it was not possible to observe this tendency and in the first molar region, an increasing tendency of bone availability in depth was observed. With increased angulation in the region of first molars, greater bone availability was found at an angulation of 75° or 90°. This increase in bone availability is probably related to the anatomy of this region. The pneumatization of the maxillary sinus decreases the bone availability of these regions (18,35,39). For the posterior region, it will probably be necessary to install smaller and/or angled control devices at the time of installation to avoid drilling into these structures. Small perforations, smaller than 2 mm, in the maxillary sinus, do not cause complications, but larger ones should be avoided (42). The insertion of the mini-implant should respect the biological spaces and the adjacent structures (roots, nerves, vessels or air spaces) (31), although scientific support exists that indicates the reversibility of some injuries caused during the use of the devices (11,43).

When the influence of age and angle interaction on bone availability in depth was evaluated, it was observed that the higher the age and the lower the variation in angulation (90°), the lower the availability in the region of the first premolar.

## Conclusions

1. The variation in the insertion angle of the TADs interferes in the bone availability when depth is considered. The maxilla anatomical variation limits the bone availability when skeletal anchorage is used.

2. According to applied methodology, it can be concluded that the region between canines and premolars accepts better vertical angular variations for TADs insertion.

3. Molar region presented poor bone availability among the roots.

4. Age influences bone availability between roots. The older the age, the lower the bone availability.
